# Cardiovascular Disease Risk of Bus Drivers in a City of Korea

**DOI:** 10.1186/2052-4374-25-34

**Published:** 2013-11-11

**Authors:** Seung Yong Shin, Chul Gab Lee, Han Soo Song, Sul Ha Kim, Hyun Seung Lee, Min Soo Jung, Sang Kon Yoo

**Affiliations:** 1 Occupational and Environmental Medicine, Chosun University Hospital, Pilmundaero 365, Dong-gu, Gwang-Ju, 501-717, Korea

**Keywords:** Cardiovascular disease, Cardiovascular risk, Professional drivers, Bus drivers

## Abstract

**Objective:**

To prevent the occurrence of CV events such as MI and stroke among professional drivers in Korea, bus drivers were compared to other occupations through the Framingham risk scoring system (FRS) or metabolic syndrome (MS) of cardiovascular disease (CVD) risk assessment methods.

**Methods:**

In October 2012, a health examination survey was conducted for 443 male bus drivers in a big city. Their CVD risk factors were compared to those of a ‘total employed’ (A group) and ‘crafts and machine operators’ (B group) extracted from Fifth Korea National Health and Nutrition Examination Survey (KNHANES, 2010) data by using FRS and MS. We calculated proportions of the CVD risk factors distribution between bus drivers and the A, B groups by the bootstrapping method. The Odds ratio (OR) between CV event risk combining MS with CHD equivalent risk of FRS and occupational factors like shift patterns and professional driving duration/age ratios (PDAR) of bus drivers was calculated through multinominal logistic regression.

**Results:**

The proportion of BMI ≥ 25 kg/m^2^ was 53.9% and waist circumference ≥ 90cm was 40.9% among bus drivers. Hypertension and MS prevalence of bus drivers was 53.3%, 49.9% which is higher than 17.6%, 22.6% in the A group and 19.7%, 23.8% in the B group respectively. OR of high CV event risk in alternate shift was 2.58 (95% CI 1.33~5.00) in comparison with double shift pattern and OR in PDAR ≥ 0.5 was 2.18 (95% CI 1.15~4.14).

**Conclusion:**

Middle aged male drivers in a big city of Korea stand a higher chance of developing CV event than other professions of the same age.

## Introduction

Cardiovascular disease (CVD) is not only the single largest cause of death, but it is also one of the compensable work-related diseases in Korea [1]. Although it is mainly caused by risk factors such as smoking, hypertension, diabetes, hyperlipidemia, obesity, physical inactivity or diet, the fact that there is some difference in morbidity and mortality of coronary heart disease (CHD) depending on the occupation means that there is also correlation between CVD and occupational factors [2,3]. In Korea, we should recognize CVD as a compensable work-related disease resulting from deaths happening due to very long labor hours. There are three types of causal or triggering factors for CV (Cardiovascular) event such as myocardial infarctions (MI) or strokes suggested. The three types are as follows: unexpected episode because of changes in working condition, extreme overwork and job stress due to being chronically overworked for long period [4]. But the problem is that Occupational Disease Award Commission of Korea Workers’ Compensation & Welfare Service is reluctant to recognize CV events in people who are professional drivers (bus, taxi or truck drivers) as a compensable disease. For their work physical work load doesn’t look heavy and their hypertension, hyperlipidemia or obesity appears to be more related to their individual life style than professional factors.

However, risk of CV event of professional drivers has already been well documented [5-11]. Professional drivers are exposed to occupational risk factors such as shift work, long working hours, loud noise, carbon monoxide, and chemical materials. All of these increase the probability of developing CVD [11]. In addition, these workers are more likely to be obese as they burn less calories due to intensity of their work activities, have a poor and irregular diet and have to work in a sitting position for a long periods every day [12]. There are many cases of substance abuse including alcohol and cigarettes as a means of easing psychological problem like anxiety and depression. They also have a high tendency towards absenteeism, job turnover, and accidents. As for bus drivers, they much more likely to develop CVD due to stressors like inadequate cabin ergonomic factors, violence from passengers, traffic congestion, inflexible running time schedule, and rotating shift patterns [11]. These occupational factors can worsen blood pressure, total cholesterol, LDL-cholesterol, HDL-cholesterol, triglyceride, diabetes, abdominal obesity (waist circumference), resulting in higher risk of experiencing CV event among professional drivers [8,13,14].

We are planning to estimate the risk of developing CVD in Korean bus drivers. If we can compare the CVD risk of bus drivers to that of other professions, then we can utilize the data to prevent bus drivers from experiencing a CV event, and reduce the social burden that can be incurred by it. This is because a CV event while driving can cause the danger to the public through secondary damage such as a serious car accident.

Of the many CVD risk assessment systems such as SCORE (European Systematic COronary Risk Evaluation), PROCAM (Prospective Cardiovascular Munster Study), the WHO/ISH (World Health Organization and International Society of Hypertension) chart, and the Framingham risk score (FRS) system, FRS is the best known and the most commonly used [15-17]. National Cholesterol Education Program’s Adult Treatment Panel III report (ATP III) notes that metabolic syndrome (MS) is a multiplex risk factor for CVD along with FRS [18]. CVD risk assessments can be used to raise public awareness of CVD which causes a significant burden of morbidity and mortality, to communicate knowledge about that risk to individuals and subgroups of the population and to motivate adherence to recommended lifestyle changes or therapies [19]. The national data from the Korean National Health and Nutrition Examination Survey (KNHANES) provides data that can compare CVD risks among different occupations [20,21]. So we can use this data (KNHANES) to compare bus drivers’ CVD risk in one big city to the national data and to develop the systems to prevent CV event of bus drivers.

## Materials and methods

### Population samples

Physicians of Occupational and Environmental Medicine surveyed 433 male bus drivers in Gwangju city to estimate CVD risk. It is estimated that there are 10 bus companies and 930 buses and about 1,200 bus drivers working for them. And we obtained data from the Fifth KNHNES (V-1, 2010) which was conducted by the Korea Centers for Disease Control and Prevention (http://knhanes.cdc.go.kr) to compare bus drivers with other occupations. The KNHANES V-1 data was composed of stratified multistage probability sampling units based on geographic area, gender, and age, which were determined based on the 3,840 household registries of Resident registration population by Korean in 2009. First, 1,842 employed males between ages 30~69 were extracted from 8,958 persons in KNHANES V-1 raw data, subsequently, 696 males labeled ‘A group’ (total employed) were randomly selected by 5-year intervals in the bus drivers’ age distribution. 221 males were labeled ‘B group’ (means ‘crafts and machine operators’ in occupational categories) were randomly selected in the same way as the ‘A group’. Sampling weights numbers of ‘A group’ equaled 4,830,156, and those of ‘B group’ equaled 1,629,428.

### Health examination survey

An intra-city bus in Gwangju runs for 2~3 hours repeatedly on a fixed route on a regular basis and it is monitored by internet systems in real time. Physical examinations for bus drivers were conducted for about 30 to 40 minutes in the employee lounge. They took place between the time when one-way run was finished and their next round-trip run began again. The time period during the day was between 11:00 to 15:00 during October of 2012. Before the physical examinations, and while they were resting, they were asked about their professional drive periods, their forms of employment and their shift patterns of duty, their working hours, and whether or not they exercise, smoke or drink alcohol. This information was gathered through questionnaires. Fifteen to twenty minutes after the questionnaires, blood pressure (BP), body weight and length were measured while they were in the stable condition. Waist circumference was measured with them standing right at the end of normal breath expiration and the measured part of the body was the middle part between the lower borders of the rib cage and the iliac crest. BP measurement was conducted through an automatic sphygmomanometer after they were seated in a chair with arm supports and reclining pad in a comfortable position. An average BP was created during analysis by measuring blood pressure again after the first BP measure. If the systolic BP was over 140 mmHg and participants wanted, BP was remeasured through a mercury sphygmomanometer to see if it coincided with the measurements of an automatic sphygmomanometer. Lastly, we measured total cholesterol (TC), HDL-cholesterol (HDL-C), LDL-cholesterol (LDL-C), triglyceride (TG), glycosylated hemoglobin A1c (HbA1c) by taking a blood sample and running the blood tests.

### Variables

Regardless of whether or not they took a dose of antihypertensive drug, we classified mean systolic and diastolic BP that was measured twice into normal, prehypertension, and hypertension according to JNC 7 criteria [22], TC, TG, HDL-C and LDL-C were divided according to NCEP-ATPIII criteria [16]. HbA1c was converted into glucose values in accordance with glucose conversion formula suggested by Nathan [23]. The types of employment were divided into regular and irregular workers and the shift patterns of duty were divided into double-shift (working 8 hours per day) and alternate-shift (work more than 12 hours every other day). Professional driving duration and age ratios (PDAR) was divided into 0.5 or more and less to compare the degrees of their professional driving exposure. We classified drivers into current smokers, ex-smokers, and non-smokers and divided them into excessive or proper drinking by categorizing excessive drinking as those who drink more than seven glasses of alcoholic beverage twice a week. They were categorized into being an exerciser only when they did it intensively for over an hour per week.

Calculated FRS was divided depending on whether or not the CHD risk equivalent was more than 20% per 10-year risk of CHD or not [24], and if three conditions of risk factors by NCEP-ATPIII criteria were met, they were treated as MS except with regard to waist circumference at 90cm which is the cut-off for abdominal obesity in Koreans [25]. We used operational definition named ‘CV event risk’ to estimate the bus drivers’ developing CV event by combining CHD risk equivalent and MS. When they came under the category of having both a CHD risk equivalent and MS, they were classified as having a ‘high risk’ of a CV event, and in the case of coming under one of the them, they were categorized into ‘medium risk’ category and if they were not included any of the two, then they were put into a ‘low risk’ category. FRS was scored according to gender, age, smoking, TC, HDL-C, and systolic BP. MS was determined in accordance with abdominal obesity, TG, HDL-C, BP, and fasting glucose [16]. In this paper, the CV event risk can be differentiated between risk factor distributions such as gender, age, smoking, BP, abdominal obesity, glucose level, and four type of lipid panels.

### Data analysis

We calculated proportions and a 95% confidence interval of bus drivers’ smoking, drinking, BMI, waist circumference, BP, TC, TG, HDL-C, LDL-C, CHD risk equivalent, MS and CV event risk by using bootstrapping method. Similarly, the proportion and 95% confidence interval of control groups, which were A and B group extracted from KNHANS V-1 were calculated by bootstrapping method considering complex sampling weights. We tested to see if there was difference in proportions between bus drivers in the A, or B groups by using a chi-square test. We also tested to see if there was a relationship between CV event risks of bus drivers and occupational factors such as employment types, shift patterns of duty and PDAR by chi-square test. Finally, we used backward stepwise multinominal logistic regression to estimate the OR of bus drivers’ CV event risks by using CV event as a dependent variable and occupational factors as independent variable like shift patterns of duty, employment types and PDAR. A p-value of less than 0.05 was regarded to be statistically significant. All statistical analyses were performed in SPSS 21.

## Results

### General characteristics of bus drivers

Among 433 bus drivers, 59.1% were aged 50–59, which was the largest age group, 79.4% were regular workers which was also the largest worker category. Irregular workers made up the remaining 20.6% of the workers. 31.4% fell into the category of professional driving for over 30 years, regular workers made up 27.3% and irregular workers made up 47.2% (Table 1). There were a greater number of older drivers with a longer driving career among irregular workers than regular workers. There were 58.9% of those with a PDAR < 0.5. In regards to types of duty, double-shift workers created 76.9% and alternate day shifts composed 23.1% (Table 1).

**Table 1:** Characteristics of male bus drivers

		**Regular**		**Irregular**		**Total**		p-value*
Age (yr)								
	< 50	107	(31.1)	16	(18.0)	123	(28.4)	0.000
	50-59	232	(67.4)	24	(27.0)	256	(59.1)	
	≥ 60	5	( 1.5)	49	(55.0)	54	(12.5)	
Work duration (yr)								
	<20	143	(41.6)	21	(23.6)	164	(37.9)	0.001
	20-29	107	(31.1)	26	(29.2)	133	(30.7)	
	≥30	94	(27.3)	42	(47.2)	136	(31.4)	
PDAR								
	<0.5	214	(62.2)	41	(46.1)	255	(58.9)	0.004
	≥0.5	130	(37.8)	48	(53.9)	178	(41.1)	
Shift pattern of duty								
	Double	328	(95.3)	5	( 5.6)	333	(76.9)	0.000
	Alternate	16	( 4.7)	84	(94.4)	100	(23.1)	
Total		344	(100.0)	89	(100.0)	433	(100.0)	

*p-value were tested by chi-square test.

PDAR, professional driving duration/age ratio.

Shift pattern, double: work 8 hours every day. Alternate: work more than 12 hours every other day.

### CVD risk factors compared between bus drivers and KNHANS groups

There was no significant difference in the distribution of smoker, excessive drinking, exercise, glucose, TC, LDL-C in the comparison between bus drivers and the A group (the total employed in KNHANS V-1) (Table 2). In contrast, bus drivers showed 53.3% of BMI ≥ 25 kg/m^2^. This was significantly higher than the measurement of 39.5% in the A group. And the ratio of TG ≥ 200 mg/dl and HDL-C < 40mg/dl, 44.6%, 36.3% in bus drivers was also higher than in the A group which was 26.5%, 20.5% respectively. When comparing waist circumference ≥ 90 cm of bus drivers, the rate was 40.9%, which was significantly higher than the measurement of 23.6% in B group (crafts and machine operators). There was no significant difference when comparing the B group regarding the distribution of other risk factors such as smoking, excessive drinking, exercise, glucose, TC, LDL-C, and HDL-C.

**Table 2:** Distribution of cardiovascular risk factors

		**BUS drivers (443)**				KNHANES*			
				**Group A (696)†**			**Group B (221)‡**		
		**%**	**95% CI**	**%**	**95% CI**	p-value*	**%**	**95% CI**	p-value*
Age	< 50	28.4	(24.2-32.8)	34.8	(28.4-42.5)	0.404	34.2	(24.3-37.7)	0.552
	50-59	59.1	(54.5-63.7)	57.5	(52.8-62.0)		56.9	(48.5-65.0)	
	≥ 60	12.5	(9.7-15.5)	7.7	(6.2-9.6)		8.9	(5.6-14.0)	
Smoking	Non	14.8	(11.8-18.0)	14.3	(11.7-17.4)	0.712	7.0	(3.8-12.4)	0.151
	Ex	43.4	(38.8-48.0)	38.3	(33.6-43.3)		41.8	(35.2-48.7)	
	Current	41.8	(37.4-46.4)	47.4	(42.9-51.9)		51.2	(44.8-57.6)	
Drinking	No/proper	74.6	(70.2-78.8)	76.1	(71.7-80.0)	0.869	75.7	(67.7-82.2)	0.920
	High risk	25.4	(21.2-29.8)	23.9	(20.0-28.3)		24.3	(17.8-32.3)	
Exercise	Yes	23.3	(19.4-27.5)	33.3	(29.4-37.5)	0.116	34.5	(27.6-42.0)	0.080
	No	76.7	(72.5-80.6)	66.7	(62.5-70.6)		65.5	(58.0-72.4)	
BMI	< 25.0	46.7	(41.8-51.3)	60.5	(56.2-64.6)	0.050	61.8	(54.3-68.8)	0.032
	≥ 25.0	53.3	(48.7-58.2)	39.5	(35.4-43.8)		38.2	(31.2-45.7)	
WC	< 90	59.1	(54.3-64.0)	72.8	(68.8-76.4)	0.057	76.4	(68.8-82.5)	0.013
	≥ 90	40.9	(36.0-45.7)	27.2	(23.6-31.2)		23.6	(17.5-31.2)	
BP	Normal	8.8	(6.2-11.5)	40.8	(35.9-45.9)	0.000	43.8	(35.7-52.2)	0.000
	Pre HTN	37.9	(33.5-42.7)	41.6	(36.7-46.7)		36.5	(28.8-44.9)	
	HTN	53.3	(48.5-57.7)	17.6	(14.2-21.5)		19.7	(14.3-26.6)	
ISH	No	67.4	(63.3-71.8)	96.2	(94.1-97.6)	0.000	97.2	(94.6-98.6)	0.000
	Yes	32.6	(28.2-36.7)	3.8	(2.4-5.9)		2.8	(1.4-5.4)	
Glucose	< 110	73.2	(69.1-77.4)	78.9	(75.1-82.3)	0.344	82.9	(76.7-87.8)	0.097
	≥ 110	26.8	(22.6-30.9)	21.1	(17.7-24.9)		17.1	(12.2-23.3)	
TC	< 200	61.4	(56.8-65.8)	57.3	(52.8-61.7)	0.822	60.5	(52.2-68.2)	0.985
	200-239	28.9	(24.2-33.3)	33.1	(28.8-37.8)		30.2	(22.9-38.7)	
	≥ 240	9.7	(6.9-12.5)	9.6	(7.2-12.6)		9.3	(5.7-14.7)	
LDL-C	< 130	74.6	(70.7-78.5)	69.8	(65.2-74.0)	0.537	68.8	(54.6-78.9)	0.285
	130-159	19.6	(15.9-23.3)	20.3	(17.0-24.0)		19.8	(11.9-31.2)	
	≥160	5.8	(3.5-8.1)	9.9	(7.2-13.6)		12.2	(5.9-23.7)	
TG	< 150	35.8	(31.2-40.6)	54.4	(49.7-58.9)	0.015	47.8	(39.5-56.2)	0.190
	150-199	19.6	(15.9-23.3)	19.1	(16.0-22.7)		18.7	(13.3-25.7)	
	≥ 200	44.6	(40.0-49.4)	26.5	(23.1-30.3)		33.5	(25.9-42.1)	
HDL-C	≥ 60	9.0	(6.2-11.8)	17.9	(14.4-21.9)	0.020	18.5	(12.6-26.2)	0.139
	59-40	54.7	(50.1-59.6)	61.6	(56.9-66.1)		51.2	(43.4-59.0)	
	< 40	36.3	(31.9-40.4)	20.5	(17.0-24.6)		30.3	(24.1-37.3)	
MS	No	50.1	(45.3-55.0)	77.4	(73.8-80.6)	0.000	76.2	(68.5-82.5)	0.000
	Yes	49.9	(45.0-54.7)	22.6	(19.4-26.2)		23.8	(17.5-31.5)	
CHD risk equivalent§	No	82.9	(79.2-86.4)	89.9	(86.8-92.3)	0.148	88.4	(82.1-92.8)	0.267
	Yes	17.1	(13.6-20.8)	10.1	(7.7-13.2)		11.6	(7.2-17.9)	
CV event risk∥	Low	45.7	(40.6-50.6)	71.1	(66.9-74.9)		67.6	(59.3-74.8)	
	Medium	41.6	(37.0-46.4)	25.1	(21.4-29.3)	0.001	29.5	(22.6-37.6)	0.004
	High	12.7	(9.7-15.9)	3.8	(2.4-6.1)		2.9	(1.1-7.3)	

p-value were tested by chi-square test, 95% CI of frequency ratio by bootstrapping method.

BMI (kg/m^2^): body mass index, WC (cm): waist circumference.

BP: blood pressure, HTN: hypertension, ISH: isolated systolic hypertension, TC (mg/dl): total cholesterol, TG (mg/dl): triglyceride, LDL-C (mg/dl): low density lipoprotein cholesterol, HDL-C (mg/dl): high density lipoprotein cholesterol, MS: metabolic syndrome.

*Weighted proportion of 2010’ Korean National Health and Nutrition Examination Survey.

†Group A: All employer (weighted number 4,830,156; unweighted case 696).

‡Group B: Crafts and machine operators(weighted number 1,629,428; unweighted case 221).

§CHD risk equivalent: >20% per 10-year of Framingham coronary heart disease risk score.

^∥^Cardiovascular disease event as stroke or myocardial infarction, High; both metabolic syndrome an CHD risk equivalent, Medium; one of the two, Low; neither metabolic syndrome nor CHD risk equivalent.

The main characteristic feature was that there was a big difference in hypertension and MS ratio. 53.3% of bus drivers experienced hypertension (BP ≥ 140/90 mmHg), which two times higher than the 17.6% in A group and 19.7% in B group. In particular, the incidence of isolated systolic hypertension (systolic BP ≥ 140 mmHg, diastolic BP < 90 mmHg) was 32.6% which was significantly higher than 3.8% in A group and 2.8% in B group. The ratio of MS was 49.9% in the bus driver group. This was more than two times higher than 22.6% in A group and 23.8% in B group. In contrast, the ratio of CHD risk equivalent was 17.1% in bus drivers and 10.1% in A group and 11.6% in B group, but there was no significant difference (Table 2).

### CV event risk and professional driving

As for high risk of CV events which came under the CHD risk equivalent and MS, the A group was 3.8%, the B group was 2.9%, and the bus drivers were 12.7%, which was three to four times higher than that of either A group and B group (Table 2). As for relationship between CV event risks and occupational factors in bus drivers, the high risk ratio of alternate in shift pattern duty was 20.2% and that in PDAR ≥ 0.5 was 17.4%, which was significantly higher than those with double shift duty or PDAR<0.5 group (Table 3). In the backward stepwise multinominal logistic regression between CV event risks and shift patterns, PDAR, and employed patterns, OR of high risk of CV events in alternate shift was 2.58 (95% CI 1.33~5.00) in comparison with double shift duty pattern, and OR in PDAR ≥ 0.5 was 2.18 (95% CI 1.15~4.14) (Table 4).

**Table 3:** CV event risk of bus drivers by occupational factors

						**CV event risk**				**P-value**
			**Low**		**Medium**		**High**		**Total**	
Employed type										
	Regular	158	(45.9)	149	(43.3)	37	(10.8)	344	(100.0)	0.044
	Irregular	40	(44.9)	31	(34.8)	18	(20.2)	89	(100.0)	
Shift Pattern										
	Double	155	(46.5)	145	(43.5)	33	(9.9)	333	(100.0)	0.005
	Alternate	43	(43.0)	35	(35.0)	22	(22.0)	100	(100.0)	
PDAR										
	<0.5	127	(49.8)	104	(40.8)	24	(9.4)	255	(100.0)	0.022
	≥0.5	71	(39.9)	76	(42.7)	31	(17.4)	178	(100.0)	

*p-value were tested by chi-square test.

PDAR, professional driving duration/age ratio.

Cardiovascular disease event as stroke or myocardial infarction.

High; both metabolic syndrome and CHD risk equivalent, Medium; one of the two, Low; neither metabolic syndrome nor CHD risk equivalent.

**Table 4:** Odds ratio of CV event risk to occupational factors

**CV event risk**	**Unadjusted**		Adjusted*	
	**OR**	**95% CI**	**OR**	**95% CI**
Medium				
Shift pattern (double vs alternative)	0.86	(0.52-1.42)	0.83	(0.53-1.38)
PDA ratio (<0.5 vs ≥0.5)	1.28	(0.85-1.94)	1.31	(0.86-1.98)
High				
Shift pattern (double vs alternative)	2.87	(1.50-5.51)	2.58	(1.33-5.00)
PDAR (<0.5 vs ≥0.5)	2.44	(1.32-4.58)	2.18	(1.15-4.14)

*Backward stepwise multinominal logistic regression, adjusted for employed type, shift duty pattern and PDAR.

PDAR, professional driving duration/age ratio.

## Discussion

For a long time, there have been many reports suggesting that professional drivers have a higher chance of developing CHD because of the greater psychiatric pressure in their working situations or their high work loads that include driving in heavy traffic, obesity, low physical activity, high demand and low decision latitude [5,26,27]. Also, some mechanisms were suggested that trigger hypertension through cardiovascular hyper-reactivity for stressors and CHD is increased [6]. But in Korea where CVD is classified as a compensable work-related disease, the fact that professional drivers stand a higher chance of developing a CV event such as MI or stroke compared to those in other occupational categories is not well recognized. That is because there has not been enough evidence for Koreans. So we used KNHANS V-1 data extracted from stratified multistage sampling of the population of Koreans to check if bus drivers have a high risk of developing a CV event as compared to total employed population in Korea. Though bus drivers in one city cannot represent all professional drivers in Korea, there might be beneficial clues to help those who are engaged as professional drivers. To start with, because 71.6% of participants in this study are men over the age of 50, we randomly extracted from KNHANS V-1(2010) data to make our comparison so that the age distribution could be similar with the constituted A group (total employed) and the B group (crafts and machine operators) during our analysis.

A characteristic feature in the result is that when it comes to risk factors such as smoking, exercise, TC, LDL-C, glucose level, there was no significant difference between drivers and the A group and the B group. The rate of TG ≥ 200 mg/dl and HDL-C < 40 mg/dl of bus drivers was relatively low compared to that of the A group and no difference compared to the B group.

But a degree of hypertension (BP ≥ 140/90 mmHg) of bus drivers was 53.3% which was about two times higher than that of A group 17.6% and B group 19.7%. There have been various types of hypertension prevalent among professional drivers. From the 1999–2004 NHANES, while overall occupational groups in 40 states showed a 21.3% occurrence rate of 28.7%, which is the highest among the same occupational groups [28]. Different papers on population and age distributions are as follows, 31.3% of express bus drivers in Korea [14], 37.2% of truck drivers in Brazil [29], 42.9% of bus and truck drivers in Iran [30]. In the comparison research of those bus drivers aged 50–60 in Taiwan and skilled workers, high prevalence was reported at an occurrence of respectively 53.6%, and 37.9% [8].

The reason why there was a high prevalence of hypertension in this study is that 70% of participants comprised of people over the age of 50 and over 40% of them had been engaged in professional driving. There may have been other interfering factors such as the time of measurement of hypertension or obesity [31]. Though blood pressure was measured during the rest period after driving, their tension might not have been completely dissipated because of that period being their break period between shifts. Driving in the city is one of the uncontrollable tasks that creates pressure due to time constraints, monotonous activities of repetitive-routine [32]. And while driving, bus drivers are faced with various many distraction factors such as radio broadcasts, ticket machines, passenger conversations or behavior, weather, bus cabin environment, road side advertising, fatigue or medications for personal condition [33]. Job strain is a risk factor for blood pressure elevation [32,34,35]. During non-work hours, they showed normal BP, but right before or after driving, and during most of the driving shift, their blood pressure tended to get higher [6]. An ambulatory BP monitoring study for taxi drivers also showed that BP during the work day was significantly higher than that during the non-work days, with a stronger effect in a hypertensive subject [36]. What was especially remarkable was that isolated systolic hypertension (ISH) is an important predictor of CHD or a stroke [37], but there was a significant difference between bus drivers and the A group and the B group. There was a possibility of white-coat hypertension being over-measured during the measurement, but masked hypertension (keeping high BP while driving) is also a possibility in that the majority of bus drivers regarded their office blood pressure as normal [38-40]. Therefore, high ISH prevalence in this middle aged bus drivers shows a higher chance of a CV event [41]. Waist circumference seems to have a strong association with the risk of hypertension [42]. In this study, we presume that the cause of high hypertension prevalence is related to obesity. The reason why bus driver showed high BMI (BMI ≥ 25 kg/m^2^) and waist circumference (WC ≥ 90cm) as compared to the control group (KNHANS V-1) is due to their irregular eating habits and low physical activity rates due to sitting on the job [11]. As abdominal visceral adipose tissue increases, fasting glucose and TG increases, HDL-C decreases, and hypertension or diabetes increases [43]. One of the mechanisms of increasing hypertension due to obesity is the hormone leptin which is derived from fat tissues and activates sympathetic nervous system [44]. That is, psychological stress, irregular eating habits and the lower physical activity level of bus drivers while driving will act as a combination factors and increase BP. As a consequence, high prevalence of hypertension is deeply related to factors such as psychological stressor or obesity.

The rate of CHD risk equivalents which is 10-year absolute risk of FRS ≥ 20% in bus drivers was higher than that of the A group and the B group, but there was no significant difference, even though MS is two times higher. Concepts of CHD risk equivalent in ATP III are designed for treating LDL-C very aggressively by preventing them proactively from developing into clinical coronary disease. MS is also for reducing CHD risk as a secondary target along with LDL-C as a primary target [16,24,45]. MS is a stronger predictor of type 2 diabetes mellitus than of CHD, but MS serves as a simple clinical tool for identifying high-risk subjects predisposed to CVD or type 2 diabetes mellitus [46]. In comparing FRS to MS using KNHANS III [21], FRS is more deeply related to MS. MS can also be defined as an independent determinant in low 10-year risk individuals [47]. Thus, in this study, we used an operational definition called ‘CV event risk’ combining CHD risk equivalent and MS to utilize age, smoking, abdominal obesity, BP, TC, TG, HDL-C, and fasting glucose which are all CVD risk factors. We categorized CV event risks into high category if they came under CHD risk equivalent and MS, and as for bus drivers it was 12.7% which was three to four times higher than those of both groups. It means that bus drivers stand a higher chance of developing a CV event compared to those in other occupational categories. There is a lot of relevant research in the relationship between professional driving and CVD [7,9,10,48-50]. Relative risk of MI of male bus drivers aged 30–74 of Stockholm in the Sweden during 1976–84 was 1.53 (95% CI 1.15-2.05) [48] and OR of MI of male bus drivers aged 45–70 during 1994–95 was 2.14 (95% CI 1.34~3.41) [9]. In a ten-year follow-up of professional drivers aged 20–59 in Denmark during 1981–1990, Standardized Hospitalization Ratios (SHR) caused by stroke was very high 130 (95% CI 123.6~137.5)[7]. Stroke SHR of bus drivers during 1994–2003, was high due to cerebral infarction rather than non-traumatic intracranial hemorrhage [10]. CV event can differ depending on a type of driving. In a five-year longitudinal study for urban bus drivers [49], there was a positive correlation between the average number of hours of bus driving per week and blood pressure. And in a study measuring brachial-ankle pulse wave velocity [50], a risk of arteriosclerosis of long-term shift drivers was higher compared to that of regular drivers and short-term shift drivers. In this study as well, in the case of PDAR ≥ 0.5 and alternate days in shift pattern of duty, high risk of CV event rate and OR increased. This means that the longer one is engaged in professional driving and the longer one’s driving hours per day are, the higher the risk of developing CVD is.

A characteristic feature in this study was that we presented the evidence that bus drivers have a higher chance of experiencing a CV event compared with 'crafts and machine operators' classified as the same occupation, or 'total employed' in Korean. In particular, hypertension prevalence was high, which means their BP can get higher while driving than resting.

But there is a limitation in cross-sectional study. Though there was a need to compare BP during the driving period, before and after driving and resting period in order to evaluate changes in BP, this was not thoroughly conducted. In addition, since a fasting state was not sustained during blood sampling, there is a possibility of rate of MS being overestimated due to changes in triglyceride values. In the future, there is a need of track observation for more elaborate CVD risk assessments.

## Competing interests

The authors declare that they have no competing interests.

## Authors’ contributions

SYS is first author. CGL is corresponding author. HSS carried out the setting of the whole survey schedule and consulting the collected data. SHK carried out the correction of indices of the metabolic syndrome, blood pressure and the occupational characteristics. HSL carried out the adjustment of measurement devices to measure the health indices such as the blood pressure, waist circumference and etc. MSJ carried out the investigation about the occupational features and other health related factors through the direct inquiry and questionnaires. SKY carried out the review and correction of the questionnaires used to this survey. All authors read and approved the final manuscript.
